# Research on Binary Mixed VOCs Gas Identification Method Based on Multi-Task Learning

**DOI:** 10.3390/s25082355

**Published:** 2025-04-08

**Authors:** Haixia Mei, Ruiming Yang, Jingyi Peng, Keyu Meng, Tao Wang, Lijie Wang

**Affiliations:** 1 Key Lab Intelligent Rehabil & Barrier Free Disable (Ministry of Education), Changchun University, Changchun 130022, China; 220401159@mails.ccu.edu.cn (R.Y.); 230401162@mails.ccu.edu.cn (J.P.); mengky@ccu.edu.cn (K.M.); 2 State Key Laboratory of Integrated Optoelectronics, College of Electronic Science and Engineering, Jilin University, Changchun 130012, China; 3 Shanghai Key Laboratory of Intelligent Sensing and Detection Technology, School of Mechanical and Power Engineering, East China University of Science and Technology, Shanghai 200237, China; wangtao@ecust.edu.cn

**Keywords:** gas sensor, multi-task learning, mixed gases, feature fusion

## Abstract

**Highlights:**

**What are the main findings?**

- A multi-task residual network (MRCA) which generates dynamic feature depending on the cross-fusion module was invented to perform VOCs gas component identification and concentration prediction.
- The dynamic weighted loss function, which can dynamically adjust the weight according to the training progress of each task.

**What is the implication of the main finding?**

- The MRCA model showed a high classification accuracy of 94.86%, as well as achieving an R^2^ score up to 0.95.
- Using only 35% of the total data length as input data leads to excellent identification performance.

**Abstract:**

Traditional volatile organic compounds (VOCs) detection models separate component identification and concentration prediction, leading to low feature utilization and limited learning in small-sample scenarios. Here, we realize a Residual Fusion Network based on multi-task learning (MTL-RCANet) to implement component identification and concentration prediction of VOCs. The model integrates channel attention mechanisms and cross-fusion modules to enhance feature extraction capabilities and task synergy. To further balance the tasks, a dynamic weighted loss function is incorporated to adjust weights dynamically according to the training progress of each task, thereby enhancing the overall performance of the model. The proposed network achieves an accuracy of 94.86% and an R^2^ score of 0.95. Comparative experiments reveal that using only 35% of the total data length as input data yields excellent identification performance. Moreover, multi-task learning effectively integrates feature information across tasks, significantly improving model efficiency compared to single-task learning.

## 1. Introduction

Volatile organic compounds (VOCs) are widely present in industrial production, vehicle emissions, and building materials, posing both short-term and long-term health risks [1,2,3,4,5]. Therefore, effective monitoring and control of VOCs are crucial for environmental protection and human health [6,7,8,9,10,11,12]. Compared to spectroscopy [13,14,15,16,17,18] and mass spectrometry [19,20,21,22], which have high equipment and environmental requirements [23], artificial olfaction technology [24] has gained widespread attention in recent years due to its miniaturization, intelligence, and low cost [25].

However, judging from the recent research trends of artificial olfaction technology, the reason why it has stagnated is due to the poor selectivity [26,27] of gas sensors, which leads to cross-sensitivity issues [28,29]. Recently, advanced artificial intelligence has been accelerating the development of artificial olfactory systems [30,31,32,33,34]. For example, Xia et al. [35] used Principal Component Analysis for feature extraction in a mixed gas classification task, achieving 96.88% accuracy with K-Nearest Neighbors (KNNs). Li et al. [36] improved gas concentration prediction by combining Variational Mode Decomposition with Extreme Learning Machine. Martono et al. [37] evaluated multiple algorithms for blood alcohol concentration prediction, with LightGBM achieving the best performance (accuracy: 0.908, F1 score: 0.617), highlighting the effectiveness of blood gas analysis for alcohol concentration estimation.

While traditional machine learning methods achieve gas detection, their reliance on manual feature extraction limits generalizability. Deep learning approaches like Convolutional Neural Network (CNN) [38] and Recurrent Neural Network (RNN) [39] have gained traction for automatic feature extraction, excelling in gas identification and concentration prediction. Chu et al. [40] transformed gas response data into grayscale images, effectively distinguishing CO and NO_2_ under varying humidity. Song et al. [41] used LASSO-RNN for mine gas concentration prediction, reducing mean squared error (MSE) (0.0029) and mean absolute error (MAE) (0.0084). Zeng et al. [42] improved mixed gas concentration regression with a dual-channel Temporal Convolutional Network (TCN), surpassing Long Short-Term Memory (LSTM), Gated Recurrent Unit (GRU), and standard TCN in prediction accuracy.

With deep learning achieving remarkable success, researchers have shifted focus toward faster and more efficient detection methods. Li et al. [43] proposed a low-cost method, which uses only 60% of rising-phase response data, enabling mixed gas concentration prediction within 10 s. However, traditional qualitative and quantitative gas analysis relies on separate systems, rendering them impractical for resource-constrained portable devices. Multi-task learning (MTL) has emerged as a promising solution. Wang et al. [44] introduced an MTL-CNN that simultaneously identifies gas types, concentrations, and states of 12 VOCs. Wang et al. [45] proposed LSTM-Attention combined with MTL (MTL-LSTMA), which achieved the classification accuracy and concentration prediction up to 98% within 30 s. Fu et al. [46] developed a real-time Progressive Prediction Algorithm integrating TCN and GRU, enabling early detection of harmful gases with enhanced speed and accuracy. Kang et al. [47] applied a multi-task CNN with a 10 s time window to classify and predict concentrations of five gases.

However, until now, there is still the challenge of improving task collaboration and feature utilization in multi-task learning. In this study, we proposed the Residual Fusion Network based on multi-task learning (MTL-RCANet) method, which aims to simultaneously perform gas identification and concentration prediction while facilitating efficient information exchange between different tasks. The method dynamically extracts local peak features from gas response data through time windows, which enhances the model’s ability to focus on key features. It introduces a channel attention mechanism and a cross-fusion module, which strengthen task collaboration by sharing information between tasks. Additionally, a dynamic weighted loss function is used to adjust the weight of each task based on its specific requirements, further improving the overall performance of the model. This approach not only improves the utilization of multi-task parameters but also provides an efficient and reliable solution for resource-constrained scenarios that require rapid identification.

## 2. Gas Experiment

**Figure 1:**
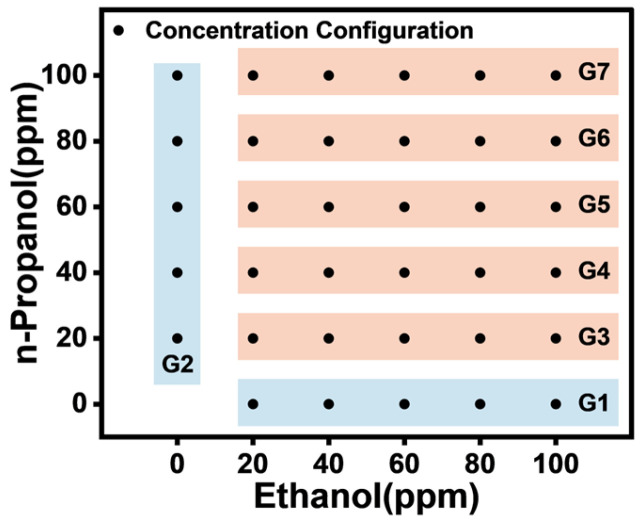
Gas experimental setup.

The sensors used in this experiment were provided by Henan Weisheng Technology, with SnO_2_ as the sensing material. Table 1 presents the gas response characteristics of each sensor. During the experiment, ethanol and n-propanol were mixed at different concentrations. As shown in Figure 1, a total of seven tests were conducted, including two single-gas response tests and five mixed-gas response tests with varying concentration ratios. Each test yielded five gas sensor response data. In the mixed-gas experiments, the concentration of one gas remained constant, while the other increased gradually from 0 to 100 parts per million (ppm) in increments of 20 ppm. G1 represents the experimental scheme for pure ethanol gas, G2 represents the experimental scheme for pure 1-propanol gas, and G3–G7 represent the experimental schemes for mixtures of ethanol and 1-propanol gases. Each point in the figure corresponds to a specific ethanol-to-1-propanol concentration ratio. Throughout the experiment, fixed time intervals were used to define the response and recovery phases in gas response testing. In the gas concentration gradient cycling response test, the sensor was exposed to the target gas for 5 min, followed by a 5 min recovery period in air. The gas sampling frequency was set to 2 Hz, and each test was repeated at least five times, resulting in a total of 175 gas sensor responses.

**Figure 2:**
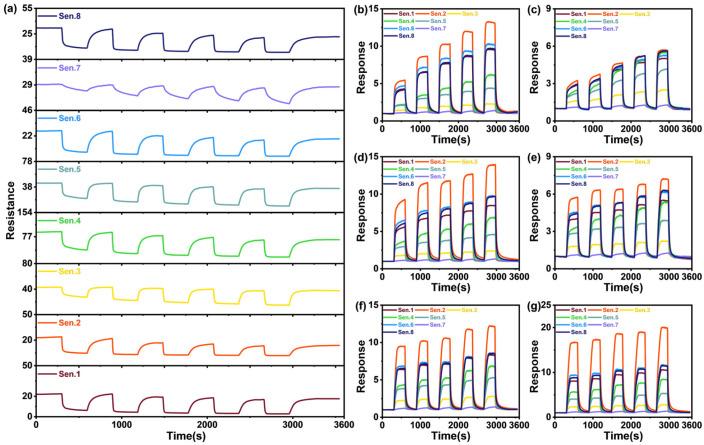
(**a**) Sensor output for single ethanol gas; (**b**) response curve for single n-propanol gas; (**c**–**g**) response curves for the mixed gases of both.

Figure 2a–g presents the time-resistance/response curves of the gas sensor array for each experimental group. Specifically, Figure 2a and Figure 2b depict the sensor array’s response to ethanol and n-propanol, respectively, while Figure 2c–g illustrate the responses to their mixed gases. The response is calculated as follows:

(1) $$s=\frac{R_{0}}{R}$$

where $R_{0}$ is the resistance of the gas sensor in air, and R is the resistance of the gas sensor in the measured gas.

## 3. Method

### 3.1. Data Preprocessing

**Figure 3:**
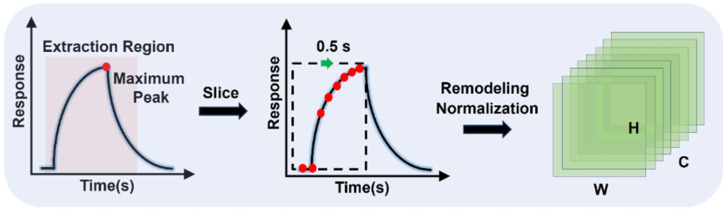
Data preprocessing flow.

As shown in Figure 3, the data preprocessing in this study involves three key stages: segmenting the response signals, selecting relevant features, and normalizing and reshaping the feature matrix.

#### 3.1.1. Response Fragment Segmentation

The gas response data undergo peak identification, where local peaks are classified based on a predefined threshold, which groups peaks within the threshold into the same category. The maximum peak from each category is then selected, which serves as a reference to segment the complete response signal. The calculation process is as follows:

The peak detection algorithm (find_peaks) is used to identify the index set $P=\left\{p_{1},p_{2},\cdots ,p_{n}\right\}$ of all local peak points in the gas response data $y_{i}$, where each $p_{j}$ satisfies the following:

(2) $$y_{p_{j}}>y_{p_{j-1}}⋀\;y_{p_{j}}>y_{p_{j+1}},j\in \left[1,n\right]$$

Calculate the distance set between adjacent peak indices, $D=\{d_{1},d_{2},\cdots ,d_{n-1}\}$, where

(3) $$d=p_{j+1}-p_{j},j\in \left[1,n-1\right]$$

Set the threshold $d_{\mathrm{threshold}}$, which is determined by the gas sampling frequency and the response recovery time of the gas sensor (i.e., the descending phase of the response curve). In the gas experiments of this study, the response recovery time of the gas sensor is 5 min (corresponding to 600 sample points). Therefore, the distance between peak point indices in each gas sensor response should be less than 600. Peak points satisfying $d<d_{\mathrm{threshold}}$ are grouped into the same cluster, forming the final grouping set G.

(4) $$G=\left\{g_{1},g_{2},\cdots ,g_{m}\right\},g=\left\{p_{k1},p_{k2},\cdots ,p_{kl}\right\},k\in \left[1,m\right]$$

For each group g, identify its corresponding maximum peak index $p_{max}$:

(5) $$p_{max}=\underset{p\in g}{\mathrm{argmax}}\;y\left[p\right]$$

The set of maximum peaks is $P_{max}=\{p_{{max}_{1}},p_{{max}_{2}},\cdots ,p_{{max}_{m}}\}$.

Using each maximum peak index $p_{max}$ as a reference, set a left offset $l_{\mathrm{offset}}$ and a right offset $r_{\mathrm{offset}}$ to determine the index range of each response segment. This results in the response segment set $S=\{s_{1},s_{2},\cdots ,s_{m}\}$. The determination strategy for $l_{\mathrm{offset}}$ and $r_{\mathrm{offset}}$ is as follows: After identifying the maximum peak point, the left side corresponds to the gas sensor’s response phase, while the right side corresponds to the recovery phase. In this study, the gas sensor’s response phase lasts for 5 min (i.e., 600 sample points). The data from the response phase will be fully utilized for feature extraction. To ensure data sufficiency, $l_{\mathrm{offset}}$ is set to 700. In the initial part of the recovery phase, we assume the presence of “numerical features” since the final response values at the end of the response vary for mixed gases with different concentration ratios. Therefore, $r_{\mathrm{offset}}$ is set to 400.

#### 3.1.2. Feature Selection

We apply a sliding time window with a step size of 0.5 s along the time axis to the segmented response fragments, which allows calculating the ratio between data variation within the window and the window width. Then, extracting the segment with the maximum ratio as the feature data. Additionally, we set the window width to a square value (e.g., n^2^) to facilitate subsequent convolution operations. The calculation process is as follows:

(6) $$R_{t}=\frac{\Delta y_{t}}{w},\Delta y_{t}=\sum_{i=t}^{t+w-1}\left(y_{i+1}-y_{i}\right)$$

Here, t is the starting position of the time window, $\Delta y_{t}$ represents the total variation within the current time window, w is the width of the time window, and $R_{t}$ is the variation rate of the window starting at position t. Among all the sliding time windows, the time segment with the maximum variation rate is selected as the feature data, and the starting position of the corresponding time window is denoted as $t_{max}$. The final feature data segment is as follows:

(7) $$y_{t_{max}}=\left\{y_{i}|t_{max}\leq i<t_{max}+w\right\}$$

#### 3.1.3. Feature Matrix Normalization and Reshaping

The response segment $y_{t_{max}}$ selected is normalized using the min-max normalization method, with the formula:

(8) $$y_{norm}=\frac{y_{t_{max}}-\min(y_{t_{max}})}{\max(y_{t_{max}})-\min(y_{t_{max}})}$$

For each gas sensor response, the response data from individual gas sensors are first reshaped into independent square feature layers using the reshape function. These layers are then stacked along the channel dimension to form multi-layer feature maps, enhancing feature extraction and analysis. As shown in Figure 3, where C represents the number of channels, H denotes the feature layer height, and W represents the feature layer width. The final gas compositions and their corresponding labels after preprocessing are shown in Table 2.

Finally, each gas sensor response was divided into five folds based on acquisition time and different test groups. One fold was used as the test set, while the remaining four folds were used as the training set, maintaining a 4:1 ratio between the training and test sets for model training and validation.

### 3.2. Multi-Task Learning Model

**Figure 4:**
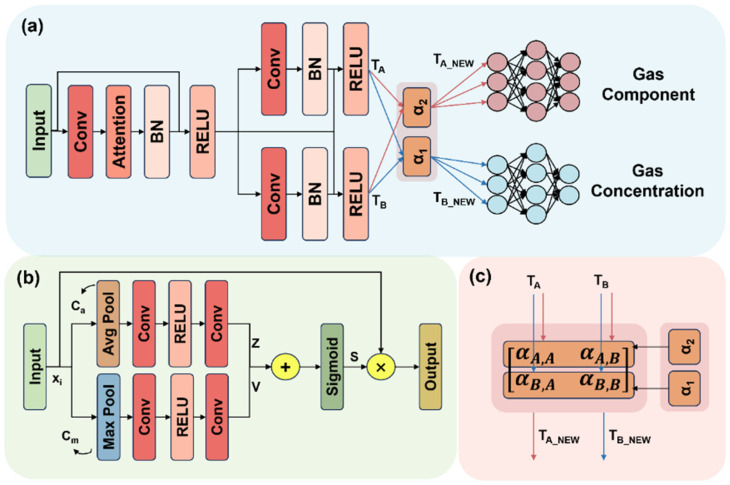
(**a**) Overall model architecture. (**b**) Channel attention mechanism module. (**c**) Cross-fusion module.

The proposed MTL-RCANet (hereafter referred to as MRCA) model, shown in Figure 4, consists of a multi-task residual network, a channel attention mechanism module, and a cross-fusion module.

#### 3.2.1. Channel Attention Mechanism

In residual networks (ResNet), the convolution operation primarily focuses on extracting deep features in spatial dimensions (H × W) but pays less attention to inter-channel dependencies (C), which may limit the network’s ability to capture channel relationships and impact feature representation [48]. To address this, we incorporate a channel attention mechanism that adaptively assigns weights to each channel based on the data’s inherent characteristics, emphasizing key channel features while suppressing secondary or irrelevant ones. The details of the channel attention mechanism are as follows:

The input to the channel attention mechanism is $X_{i}$, with a shape of C × H × W, and it consists of two branches. The first branch is retained and will be weighted after the second branch has been computed, which highlights the key channel features. In the second branch, the global average value $C_{a}$ and the maximum value $C_{m}$ are computed separately for each channel.

(9) $$C_{a}\left(c\right)=\frac{1}{H\times W}\sum_{i=1}^{H}\sum_{j=1}^{W}X_{i}\left(c,i,j\right),\forall c\in \left[1,C\right]$$

(10) $$C_{m}\left(c\right)={max}_{i=1,j=1}^{H,W}X_{i}\left(c,i,j\right),\forall c\in \left[1,C\right]$$

After global average pooling and global max pooling, two 1 × 1 convolutions are applied, with a ReLU activation layer in between, to compress and aggregate features at the channel level, learning the correlation between channels. The final outputs are Z and V.

(11) $$Z={Conv}_{2}\left(ReLU\left({Conv}_{1}\left(C_{a}\right)\right)\right)$$

(12) $$V={Conv}_{2}\left(ReLU\left({Conv}_{1}\left(C_{m}\right)\right)\right)$$

The two sub-branches, Z and V, are summed and passed through a Sigmoid activation function to output the final weight S. The weight S is then element-wise multiplied with $X_{i}$ to produce the output.

(13) $$S=Sigmoid\left(Z+V\right)$$

(14) $$Output=S⊙X_{i}$$

#### 3.2.2. Cross-Fusion Module

A key challenge in multi-task learning is how to effectively sharing beneficial weight information between tasks to enhance model performance. A typical hard parameter sharing strategy directly shares network weights. However, this approach can be unstable, with interfering weights dominating between tasks. In contrast, the cross-fusion module employs a soft parameter sharing strategy, which facilitates dynamic weight sharing that enables more flexible coordination of information flow between tasks [49]. Its core concept is to dynamically adjust the degree of feature sharing between tasks using a learnable weighting mechanism, allowing efficient fusion of task features. The fusion formula is as follows:

(15) $$\left[\begin{array}{c} T_{A}^{\prime }\\ T_{B}^{\prime } \end{array}\right]=\left[\begin{array}{cc} \alpha_{A,A} & \alpha_{A,B}\\ \alpha_{B,A} & \alpha_{B,B} \end{array}\right]\left[\begin{array}{c} T_{A}\\ T_{B} \end{array}\right]$$

Here, $T_{A}$ and $T_{B}$ are the feature maps after the second convolution for the gas component identification and concentration prediction tasks (as shown in Figure 3). $T_{A}^{\prime }$ and $T_{B}^{\prime }$ are the new feature maps after the fusion of the two tasks. The learnable matrix α contains $\alpha_{A,A}$ and $\alpha_{B,B}$, which represent the self-preserved feature weights for tasks A and B, respectively. $\alpha_{A,B}$ and $\alpha_{B,A}$ are the contribution weights from task B to task A and from task A to task B, respectively. The matrix α is a learnable parameter that is dynamically adjusted based on the data, with its initial state as the identity matrix.

#### 3.2.3. Multi-Task Residual Network

Residual neural networks have inherent advantages in parallel execution of multiple outputs and tasks, enabling them to handle various task requirements based on shared features. Additionally, through residual connections, they effectively preserve and propagate feature information, improving the model’s training efficiency. In the backbone network (excluding the channel attention mechanism and cross-fusion modules), all convolution operations use a 3 × 3 kernel size, a stride of 1, and a padding size of 2, with ReLU as the activation function. The design process for each layer is as follows:

1. Input Layer: After preprocessing, the shape of the gas response data is 8 × H × W (where H = W). The number eight represents the number of input feature maps corresponding to the number of gas sensors in the array, and H × W refers to the height and width of the feature maps.
2. Convolutional Layer: In the backbone structure of the multi-task residual network, the convolution kernel is set to the common 3 × 3 size. To avoid information loss at the edges of the feature map due to convolution, the padding size is set to two, ensuring that edge regions fully participate in feature extraction. The main purpose of convolution is to extract deeper features, so after each convolution operation, the number of channels doubles compared to the previous layer. For example, after the second convolution, the number of channels increases to 32, gradually enhancing the network’s expressive power.
3. Batch Normalization and Activation Function: To accelerate model convergence, batch normalization is applied after each convolution operation to standardize intermediate feature distributions. Since the length of the gas response data samples is relatively short, pooling and dropout operations are omitted, but batch normalization helps reduce overfitting. The ReLU activation function is chosen to improve the model’s non-linear representation and reduce computational complexity.
4. Fully Connected Layer: After completing feature extraction and fusion for tasks A and B, the feature maps are flattened and passed through three fully connected layers for transformation. These layers gradually compress and map the high-dimensional feature space, enhancing the model’s ability to represent the target task. Finally, task A outputs gas component recognition results using the Softmax function to calculate the probability distribution for each category, while task B predicts the concentrations of the two gases.

## 4. Experimental Results and Analysis

### 4.1. Hyperparameter Settings

Table 3 presents the hyperparameters of the backbone network, including a batch size of 5. Specifically, the table defines several key parameters: Map represents the number of channels in the output feature maps generated by the convolutional operations; K denotes the kernel size used in the convolution; S refers to the stride applied during convolution; P indicates the padding around the feature maps during convolution; D corresponds to the channel dimension in batch normalization. In addition, T_A_ and T_B_ represent the gas component recognition and concentration prediction tasks, respectively. The term “1st Convolutional” refers to the convolutional layer within the first residual block, whereas “2.1st–2.2nd Convolutional” refers to the convolutional layers within the second residual block, which are dedicated to handling tasks T_A_ and T_B_. This setup ensures that each task is processed with specific configurations to optimize their individual performances within the network.

### 4.2. Model Training and Validation

All experimental results are the averages from 5-fold cross-validation. The gas component recognition task is a three-class classification, and the gas concentration prediction task is a two-variable regression, each with distinct loss functions: cross-entropy loss for recognition and MSE for prediction. Since the values of cross-entropy loss lie in the range [0, 1], whereas MSE has no upper limit, the numerical difference between the two loss functions is large. A simple addition would cause the cross-entropy loss to have little impact on the final loss. Therefore, this study proposed a learnable dynamic weighting loss function to balance the numerical differences between the various losses. Three learnable parameters are defined: $\sigma_{p}$ for the propane concentration prediction, $\sigma_{e}$ for the ethanol concentration prediction, and $\sigma_{ci}$ for the gas component recognition. These parameters can be dynamically optimized through neural network gradient descent. The total loss function is defined as follows:

(16) $$TotalLoss=\frac{1}{2\sigma_{p}^{2}}\cdot {Loss}_{p}+\frac{1}{2\sigma_{e}^{2}}\cdot {Loss}_{e}+\frac{1}{\sigma_{ci}^{2}}\cdot {Loss}_{ci}+\log_{10}\left(\sigma_{p}\sigma_{e}\sigma_{ci}\right)$$

**Figure 5:**
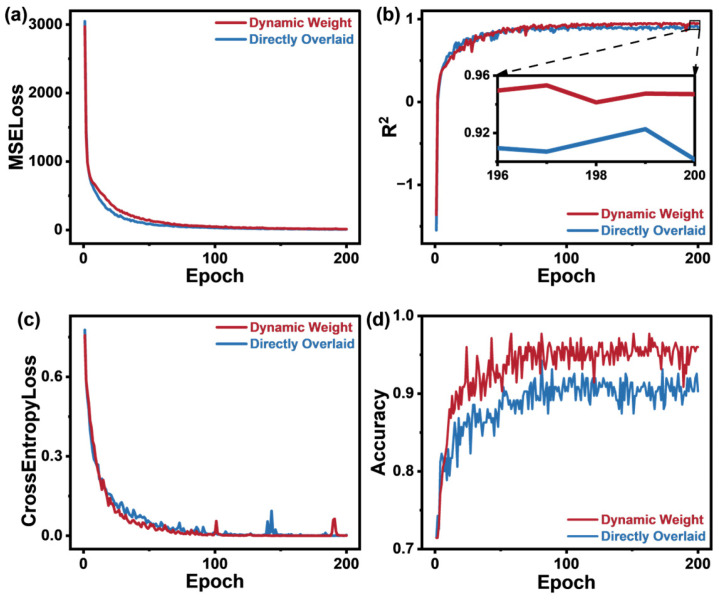
(**a**) Overall MSE loss for both gases during the training process; (**b**) overall R^2^ value for both gases during the validation process; (**c**) cross-entropy loss for gas component recognition during the training process; (**d**) accuracy of gas component recognition during the validation process.

In the above formula, TotalLoss is the final total loss, ${Loss}_{p}$ is the training loss for propane concentration prediction, ${Loss}_{e}$ is the training loss for ethanol concentration prediction, and ${Loss}_{ci}$ is the training loss for gas component recognition. This adaptive mechanism addresses the large numerical differences between the loss values, allowing for effective optimization of all tasks during training. The term $\log_{10}\left(\sigma_{p}\sigma_{e}\sigma_{ci}\right)$ acts as a regularizer, preventing $\sigma_{p}$, $\sigma_{e}$, and $\sigma_{e}$ from growing or shrinking excessively, thus ensuring model stability. Figure 5 shows the loss curves and evaluation metrics during the training and validation processes, comparing the dynamic weighted loss function with the direct sum of individual losses.

Figure 5a,b show that while directly summing individual losses leads to faster convergence, the R^2^ score on the validation set is about four percentage points lower than with the dynamic weighted loss function. The dynamic weighted loss function accelerates convergence and improves accuracy by approximately five percentage points compared to direct summation, as shown in Figure 5c,d.

### 4.3. Model Performance

**Figure 6:**
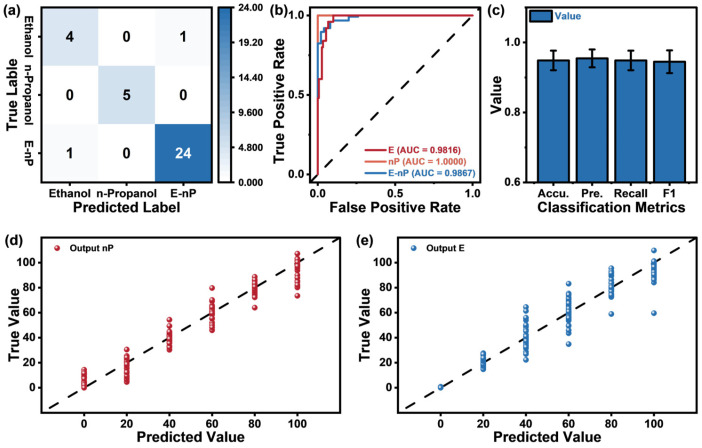
(**a**) Confusion matrix for the component recognition task; (**b**) ROC curve for each gas component classification; (**c**) accuracy, precision, recall, and F1 score for the classification task; (**d**) comparison between the true and predicted values for propanol; (**e**) comparison between the true and predicted values for ethanol.

The experimental results for gas component identification and concentration prediction are shown in Figure 6. Through 5-fold cross-validation, the model achieved 94.86% classification accuracy and an R^2^ score of 0.95 for the regression task. Table 4 presents the key evaluation metrics for both tasks, along with their standard errors.

Figure 6a presents the confusion matrix for the classification task. On average, only 2 out of 35 validation samples per fold are misclassified, while the remaining samples are correctly identified. Figure 6b displays the Receiver Operating Characteristic (ROC) curves for each gas component, with all gas types achieving an area under the curve exceeding 0.98, indicating excellent classification performance. Figure 6c summarizes the accuracy, precision, recall, and F1 score of the classification task, which are 94.86%, 95.45%, 94.86%, and 0.94, respectively. Figure 6d,e illustrate scatter plots comparing the true and predicted values for propanol and ethanol under five-fold cross-validation. Some data points exhibit noticeable deviations, which we attribute to data drift occurring during long-term sample collection.

**Figure 7:**
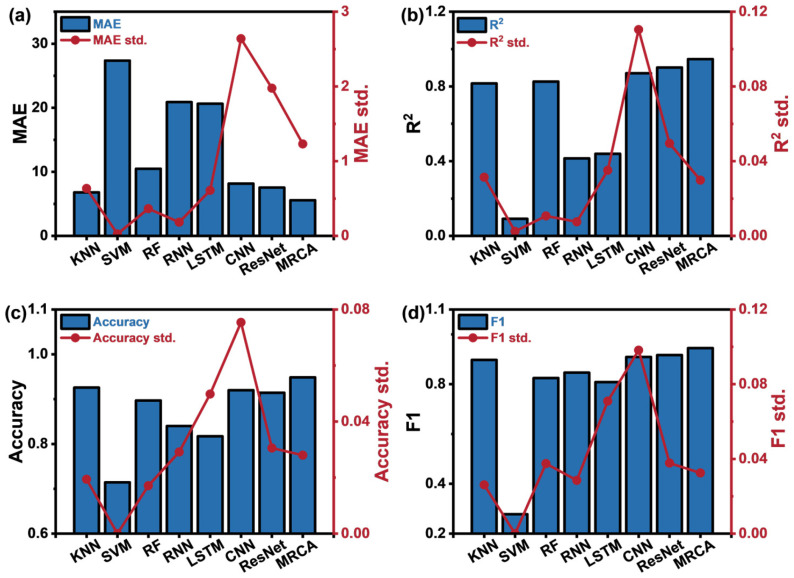
Performance evaluation metrics of eight algorithms in 5-fold cross-validation: (**a**) MAE, (**b**) R^2^ score, (**c**) accuracy, and (**d**) F1 score, with the mean values and standard errors.

To evaluate the performance differences between the proposed model and baseline models, we conducted a systematic comparison with various mainstream machine learning and deep learning models. The baseline models include traditional machine learning methods (such as KNN, Support Vector Machine (SVM), and Random Forest (RF)) as well as deep neural networks (such as RNN, LSTM, CNN, and ResNet). Experimental results indicate that the proposed model surpasses all baseline models in both classification accuracy and regression prediction precision. The corresponding results are presented in Figure 7, while Table 5 provides a detailed comparison of each model’s performance.

To validate the effectiveness of multi-task learning in jointly performing gas component identification and concentration prediction, we removed the cross-fusion module from the MRCA model and divided it into two separate networks: MRCA-C for gas component identification and MRCA-R for concentration prediction. Each network was trained independently on its respective task. The performance comparison among the three models is presented in Table 6.

**Figure 8:**
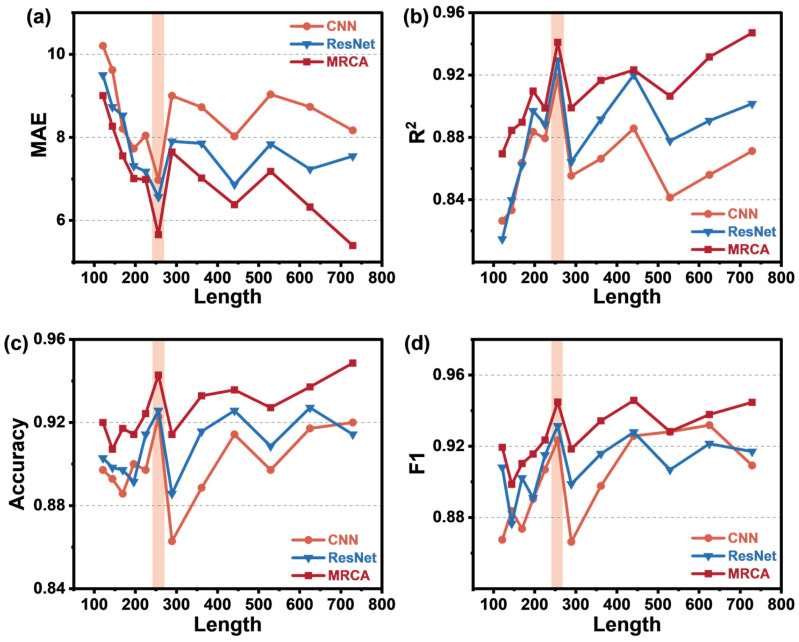
(**a**–**d**) MAE, R^2^ score, classification accuracy, and F1 score of the three algorithms: CNN, ResNet, and MRCA at different sample lengths.

To determine the optimal training sample length, we set different time window widths, 11, 12, 13, 14, 15, 16, 17, 19, 21, 23, 25, and 27 squared, and conducted comparison experiments using CNN, ResNet, and the MRCA network. The results show that when the sample length is 16^2^ (i.e., 256 data points), the model performance is comparable to that of the data with a sample length of 27^2^ (i.e., 729 data points). This indicates that appropriately shortening the sample length not only does not significantly reduce the model’s prediction accuracy but can actually improve the model’s running efficiency. The experimental results are shown in Figure 8. The pink area represents the evaluation metrics corresponding to the optimized sample length.

### 4.4. Ablation Experiment

To evaluate the contribution of each module to the model’s performance, we conducted ablation experiments by sequentially removing or modifying key modules. The three core modules examined were the dynamic weighted loss function, the channel attention mechanism, and the cross-fusion module. We hypothesize that the channel attention mechanism enhances the model’s focus on critical features, the cross-fusion module improves feature layer interaction and fusion, and the dynamic weighted loss function facilitates gradient descent optimization. To verify these hypotheses, we designed the following experiments:

1. MRCA-1: The dynamic weighted loss function’s weight parameter σ is initialized based on experience to evaluate the impact of weight initialization on model performance.
2. MRCA-2: The dynamic weighted loss function’s weight parameter σ is not initialized, aiming to evaluate the impact of not initializing the weights on model performance.
3. MRCA-3: The total loss is calculated by directly adding the individual losses to evaluate the impact of the dynamic weighted loss function on model performance.
4. NO Attention: The channel attention mechanism module is removed to evaluate its impact on model performance.
5. NO Cross: The cross-fusion module is removed to evaluate its contribution.
6. BaseLine: The baseline model, which removes both the channel attention mechanism and the cross-fusion module.

**Figure 9:**
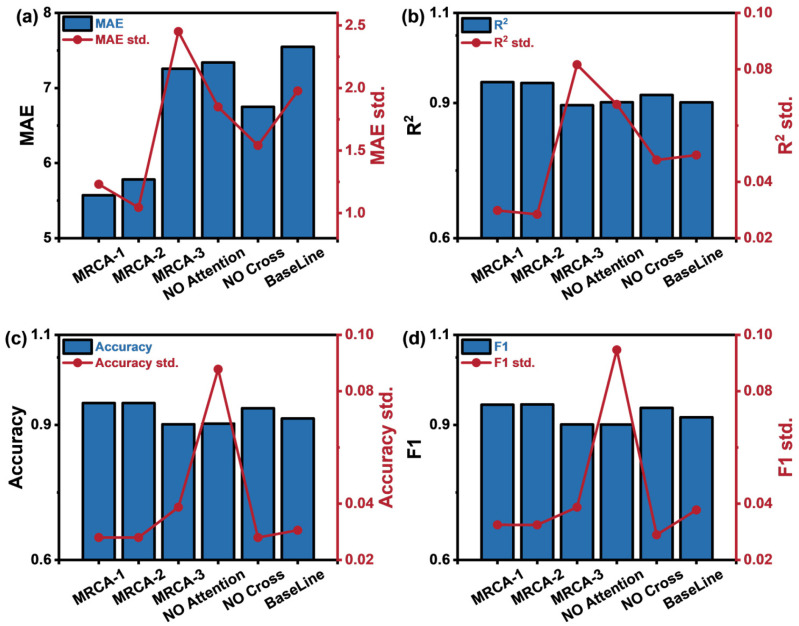
Ablation experiment performance evaluation metrics: (**a**) MAE, (**b**) R^2^ score, (**c**) accuracy, (**d**) average F1 score, and standard error.

The experimental results are shown in Figure 9, and specific experimental performances are provided in Table 7.

The experimental results show that the performance difference between using experience-initialized weight parameters (σ) and uninitialized weight parameters (σ) in the dynamic weighted loss function is minimal. This suggests that the dynamic weighted loss function is less sensitive to initial weights and allows the model to adaptively optimize loss weights, thereby simplifying the initialization process. In contrast, the MRCA-3 model, which lacks the dynamic weighted loss function, performs poorly in both classification and regression tasks.

When either the channel attention mechanism or the cross-fusion module is removed individually, model performance declines. However, when both modules are removed, performance deteriorates significantly, with the R^2^ score dropping by approximately 6% points and accuracy decreasing by 5% points.

These ablation results highlight the positive impact of the channel attention mechanism and the cross-fusion module on model performance. Additionally, the dynamic weighted loss function not only optimizes performance but also improves training efficiency.

## 5. Conclusions

This study proposed the MTL-RCANet, which simultaneously achieves a classification accuracy of 94.86% and an R^2^ score of 0.95 for concentration prediction. Compared to single-task models, MTL-RCANet significantly improves the performance of both classification and regression tasks by efficiently extracting and sharing key feature information across tasks. Additionally, a dynamic weighted loss function was introduced to address the varying loss requirements of different tasks during training. By dynamically adjusting the loss weight for each task based on its importance and difficulty, the model’s overall performance is further enhanced. Therefore, our approach not only accelerates training convergence but also effectively balances the training processes of different tasks. Further research indicates that the model can still perform detection tasks efficiently, even with just 35% of the gas response data.

In summary, the proposed method offers a new solution for gas detection tasks in fast detection and low-resource consumption scenarios, which shows great application potential. Future work can further optimize the network structure to enhance task collaboration, particularly in more complex gas mixtures or dynamic response scenarios.

## Figures and Tables

**Table 1:** Sensor model and corresponding response gases.

Model Number	Response Gas
MQ-2	Liquefied Gas, C_3_H_8_, H_2_
MQ-3	C_2_H_5_OH
MQ-4	CH_4_
MQ-5	C_4_H_10_, C_3_H_8_, CH_4_
MQ-6	C_3_H_8_, C_4_H_10_
MQ-7	CO
MQ-8	H_2_
MQ-9	CO

**Table 2:** Gas composition.

Gas Type	Single n-Propanol	Single Ethanol	n-Propanol and Ethanol
Label	01	10	11

**Table 3:** Hyperparameter list of the backbone network.

Layer	Configuration	Input Shape
1st Convolutional	Map: 16, K: 3, S: 1, P: 2	8 × H × W
CA Module	/	16 × H × W
BN, Activation	D:16, ReLU	/
2.1st–2.2st Convolutional	Map: 32, K: 3, S: 1, P: 2	/
BN, Activation	D:32, ReLU	32 × H × W
FC1	32 × H × W, 128	/
FC2	128, 64	128
FC3	64, 3 (T_A_) || 2 (T_B_)	64
Output	T_A_: 5 × 3, T_B_: 5 × 2	/

**Table 4:** Model evaluation metrics.

Accuracy	Std.	F1	Std.	MAE	Std.	R^2^	Std.
94.86%	0.03	0.94	0.03	5.40	1.26	0.95	0.03

**Table 5:** Classification and regression performance of different models.

Algo.	MAE	R^2^	Accuracy	F1
KNN	6.8000	0.8164	0.9257	0.8975
SVM	27.3700	0.0914	0.7143	0.2778
RF	10.4900	0.8263	0.8971	0.8249
RNN	20.8944	0.4151	0.8400	0.8463
LSTM	20.6333	0.4397	0.8171	0.8086
CNN	8.1681	0.8712	0.9200	0.9092
ResNet	7.3927	0.8882	0.8914	0.8984
MRCA	5.3961	0.9471	0.9486	0.9449

**Table 6:** Performance comparison between multi-task and single-task models.

Algo.	MAE	R^2^	Accuracy	F1
MRCA-C	/	/	0.9142	0.9068
MRCA-R	6.7737	0.9182	/	/
MRCA	5.3961	0.9471	0.9486	0.9449

**Table 7:** Ablation experiment.

Algo.	MAE	R^2^	Accuracy	F1
MRCA-1	5.3961	0.9471	0.9486	0.9449
MRCA-2	5.7812	0.9445	0.9486	0.9456
MRCA-3	7.2567	0.8953	0.9014	0.9011
NO Attention	7.3411	0.9017	0.9029	0.9009
NO Cross	6.7503	0.9179	0.9371	0.9378
BaseLine	7.3927	0.8882	0.8914	0.8984

## Data Availability

Data are contained within the article.

## References

[1] Li P., Wang C., Li L., Zheng T. (2024). Bioaerosols and VOC emissions from landfill leachate treatment processes: Regional differences and health risks. J. Hazard. Mater..

[2] Kim S.J., Lee S.J., Hong Y., Choi S.D. (2024). Investigation of priority anthropogenic VOCs in the large industrial city of ulsan, south korea, focusing on their levels, risks, and secondary formation potential. Atmos. Environ..

[3] Miao G., Wang Y., Wang B., Yu H., Liu J., Pan R., Zhou C., Ning J., Zheng Y., Zhang R. (2023). Multi-omics analysis reveals hepatic lipid metabolism profiles and serum lipid biomarkers upon indoor relevant VOC exposure. Environ. Int..

[4] Wang H., Zhang R., Kong H., Wang K., Sun L., Yu X., Zhao J., Xiong J., Tran P.T.M. (2024). Balasubramanian. long-term emission characteristics of VOCs from building materials. J. Hazard. Mater..

[5] Zhou L., Huang C., Lu R., Wang X., Sun C., Zou Z. (2024). Associations between VOCs and childhood asthma in Shanghai, China: Impacts of daily behaviors. Atmos. Pollut. Res..

[6] Hussain M.S., Gupta G., Mishra R., Patel N., Gupta S., Alzarea S.I., Kazmi I., Kumbhar P., Disouza J., Dureja H. (2024). Unlocking the secrets: Volatile organic compounds (VOCs) and their devastating effects on lung cancer. Pathol.-Res. Pract..

[7] Peng J., Mei H., Yang R., Meng K., Shi L., Zhao J., Zhang B., Xuan F., Wang T., Zhang T. (2024). Olfactory diagnosis model for lung health evaluation based on pyramid pooling and shap-based dual encoders. ACS Sens..

[8] Song J., Li R., Yu R., Zhu Q., Li C., He W., Liu J. (2024). Detection of VOCs in exhaled breath for lung cancer diagnosis. Microchem. J..

[9] Liu H., Fang C., Zhao J., Zhou Q., Dong Y., Lin L. (2024). The detection of acetone in exhaled breath using gas pre-concentrator by modified metal-organic framework nanoparticles. Chem. Eng. J..

[10] Lv E., Wang T., Yue X., Wang H., Zeng J., Shu X., Wang J. (2024). Wearable SERS sensor based on bionic sea urchin-cavity structure for dual in-situ detection of metabolites and VOCs gas. Chem. Eng. J..

[11] Mei H., Peng J., Wang T., Zhou T., Zhao H., Zhang T., Yang Z. (2024). Overcoming the limits of cross-sensitivity:pattern recognition methods for chemiresistive gas sensor array. Nano-Micro Lett..

[12] Raina S., Bharti A., Singh H.M., Kothari R., Tyagi V.V., Pathania D., Buddhi D., Yadav B.C., Kumar P. (2024). Chapter 1—Applications of gas and VOC sensors for industry and environmental monitoring: Current trends and future implications. Complex and Composite Metal Oxides for Gas, VOC, and Humidity Sensors.

[13] Fan H., Wang P., Zhang H., Hu M., Zhu C., Wang Q. (2024). Zero-absorption-assisted multitask learning for simultaneous measurement of acetylene concentration and gas pressure from overlap-deformed spectra. Opt. Laser Technol..

[14] Gong Z., Fan Y., Guan Y., Wu G., Mei L. (2024). Empirical Modal Decomposition Combined with Deep Learning for Photoacoustic Spectroscopy Detection of Mixture Gas Concentrations. Anal. Chem..

[15] Hou J., Liu X., Sun H., He Y., Qiao S., Zhao W., Zhou S., Ma Y. (2025). Dual-Component Gas Sensor Based on Light-Induced Thermoelastic Spectroscopy and Deep Learning. Anal. Chem..

[16] Kan Z., Zhang Y., Luo L., Cao Y. (2024). Ultraviolet absorption spectrometry with symmetrized dot patterns and deep learning for quantitative analysis of SO2, H2S, CS2 mixed gases. Eng. Appl. Artif. Intell..

[17] Kistenev Y.V., Skiba V.E., Prischepa V.V., Borisov A.V., Vrazhnov D.A. (2024). Gas-mixture IR absorption spectra denoising using deep learning. J. Quant. Spectrosc. Radiat. Transf..

[18] Zhou Y., Jiang M., Dou W., Meng D., Wang C., Wang J., Wang X., Sun L., Jiang S., Chen F. (2023). Narrow-band multi-component gas analysis based on photothermal spectroscopy and partial least squares regression method. Sens. Actuators B Chem..

[19] Chakraborty P., Borras E., Rajapakse M.Y., McCartney M.M., Bustamante M., Mitcham E.J., Davis C.E. (2023). Non-destructive method to classify walnut kernel freshness from volatile organic compound (VOC) emissions using gas chromatography-differential mobility spectrometry (GC-DMS) and machine learning analysis. Appl. Food Res..

[20] Dwyer D.B., Niedziela J.L., Miskowiec A. (2025). Tandem pyrolysis evolved gas–gas chromatography–mass spectrometry. J. Anal. Appl. Pyrolysis.

[21] Rahmani N., Mani-Varnosfaderani A. (2024). Profiling volatile organic compounds of different grape seed oil genotypes using gas chromatography-mass spectrometry and chemometric methods. Ind. Crops Prod..

[22] Zhang Z., Zhang Q., Xi Y., Zhou Y., Zhan M. (2024). Establishment of a headspace-thermal desorption and gas chromatography-mass spectrometry method (HS-TD-GC-MS) for simultaneous detection of 51 volatile organic compounds in human urine: Application in occupational exposure assessment. J. Chromatogr. A.

[23] Zhao Y., Liu Y., Han B., Wang M., Wang Q., Zhang Y.-n. (2023). Fiber optic volatile organic compound gas sensors: A review. Coord. Chem. Rev..

[24] Bing Y., Zhang F., Han J., Zhou T., Mei H., Zhang T. (2023). A method of ultra-low power consumption implementation for MEMS gas sensors. Chemosensors.

[25] Chen H., Huo D., Zhang J. (2022). Gas Recognition in E-Nose System: A Review. IEEE Trans. Biomed. Circuits Syst..

[26] Li J., Zhao H., Wang Y., Zhou Y. (2024). Approaches for selectivity improvement of conductometric gas sensors: An overview. Sens. Diagn..

[27] Yin X.-T., Dastan D., Gity F., Li J., Shi Z., Alharbi N.D., Liu Y., Tan X.-M., Gao X.-C., Ma X.-G. (2023). Gas sensing selectivity of SnO2-xNiO sensors for homogeneous gases and its selectivity mechanism: Experimental and theoretical studies. Sens. Actuators A Phys..

[28] Panda S., Mehlawat S., Dhariwal N., Kumar A., Sanger A. (2024). Comprehensive review on gas sensors: Unveiling recent developments and addressing challenges. Mater. Sci. Eng. B.

[29] Wang Z., Li Y., He X., Yan R., Li Z., Jiang Y., Li X. (2024). Improved deep bidirectional recurrent neural network for learning the cross-sensitivity rules of gas sensor array. Sens. Actuators B Chem..

[30] Pan X., Chen J., Wen X., Hao J., Xu W., Ye W., Zhao X. (2023). A comprehensive gas recognition algorithm with label-free drift compensation based on domain adversarial network. Sens. Actuators B Chem..

[31] Wei G., Xu Y., Lv X., Jiao S., He A. (2024). An adaptive drift compensation method based on integrated dual-channel feature fusion for electronic noses. IEEE Sens. J..

[32] Yao Y., Chen B., Liu C., Qu C. (2023). Investigation on the combined model of sensor drift compensation and open-set gas recognition based on electronic nose datasets. Chemom. Intell. Lab. Syst..

[33] Laref R., Losson E., Sava A., Adjallah K., Siadat M. A comparison between SVM and PLS for E-nose based gas concentration monitoring. Proceedings of the 2018 IEEE International Conference on Industrial Technology (ICIT).

[34] Xu W., Tang J., Xia H., Sun Z. Prediction method of dioxin emission concentration based on PCA and deep forest regression. Proceedings of the 2021 40th Chinese Control Conference (CCC).

[35] Xia W., Song T., Yan Z., Song K., Chen D., Chen Y. A method for recognition of mixed gas composition based on PCA and KNN. Proceedings of the 2021 19th International Conference on Optical Communications and Networks (ICOCN).

[36] Li K., Yang G., Wang K., Lu B., Jia J., Sun T. Prediction of dissolved gases concentration in transformer oil based on VMD and ELM. Proceedings of the 2023 IEEE 7th Conference on Energy Internet and Energy System Integration (EI2).

[37] Martono N.P., Kuramaru S., Igarashi Y., Yokobori S., Ohwada H. Blood alcohol concentration screening at emergency room: Designing a classification model using machine learning. Proceedings of the 2023 14th International Conference on Information & Communication Technology and System (ICTS).

[38] Zhu R., Gao J., Li M., Gao Q., Wu X., Zhang Y. (2023). A ppb-level online detection system for gas concentrations in CS2/SO2 mixtures based on UV-DOAS combined with VMD-CNN-TL model. Sens. Actuators B Chem..

[39] Mao G., Zhang Y., Xu Y., Li X., Xu M., Zhang Y., Jia P. (2023). An electronic nose for harmful gas early detection based on a hybrid deep learning method H-CRNN. Microchem. J..

[40] Chu J., Li W., Yang X., Wu Y., Wang D., Yang A., Yuan H., Wang X., Li Y., Rong M. (2021). Identification of gas mixtures via sensor array combining with neural networks. Sens. Actuators B Chem..

[41] Song S., Chen J., Ma L., Zhang L., He S., Du G., Wang J. (2023). Research on a working face gas concentration prediction model based on LASSO-RNN time series data. Heliyon.

[42] Zeng L., Xu Y., Ni S., Xu M., Jia P. (2023). A mixed gas concentration regression prediction method for electronic nose based on two-channel TCN. Sens. Actuators B Chem..

[43] Li X., Guo J., Xu W., Cao J. (2023). Optimization of the mixed gas detection method based on neural network algorithm. ACS Sens..

[44] Wang T., Zhang H., Wu Y., Jiang W., Chen X., Zeng M., Yang J., Su Y., Hu N., Yang Z. (2022). Target discrimination, Concentration prediction of binary mixed gases based on random forest algorithm in the electronic nose system mixed gases, and status judgment of electronic nose system based on large-scale measurement and multi-task deep learning. Sens. Actuators B Chem..

[45] Wang X., Zhao W., Ma R., Zhuo J., Zeng Y., Wu P., Chu J. (2024). A novel high accuracy fast gas detection algorithm based on multi-task learning. Measurement.

[46] Fu C., Zhang K., Guan H., Deng S., Sun Y., Ding Y., Wang J., Liu J. (2024). Progressive prediction algorithm by multi-interval data sampling in multi-task learning for real-time gas identification. Sens. Actuators B Chem..

[47] Kang M., Cho I., Park J., Jeong J., Lee K., Lee B., Del Orbe Henriquez D., Yoon K., Park I. (2022). High accuracy real-time multi-gas identification by a batch-uniform gas sensor array and deep learning algorithm. ACS Sens..

[48] Zhang S., Cheng Y., Luo D., He J., Wong A.K.Y., Hung K. (2021). Channel attention convolutional neural network for chinese baijiu detection with E-Nose. IEEE Sens. J..

[49] Misra I., Shrivastava A., Gupta A., Hebert M. Cross-stitch networks for multi-task learning. Proceedings of the 2016 IEEE Conference on Computer Vision and Pattern Recognition (CVPR).

